# Supplementary material for: The Use of microRNAs in the Management of Endometrial Cancer: A Meta-Analysis
Source: Cancers (Basel). 2019 Jun 16;11(6):832. doi: 10.3390/cancers11060832 (PMC6628044; doi:10.3390/cancers11060832)
Supplement: Supplementary file 1 [file cancers-11-00832-s001.pdf]

Supplementary Materials: The Use of microRNAs in the Management of Endometrial Cancer: A Meta-Analysis

Romain Delangle, Tiphaine De Foucher, Annette K. Larsen, Michèle Sabbah, Henri Azaïs, Sofiane Bendifallah, Emile Daraï, Marcos Ballester, Céline Mehats, Catherine Uzan and Geoffroy Canlorbe

Table S1. miRs Involved in Endometrial Cancer.

Name of the miR	Increased Abundance in Tumor Tissue or Associated with Poor Prognosis	Abundance Decreased in Tumor Tissue or Associated with a Better Prognosis	Reference
miR-1		X	[1]
miR-1-1		X	[2]
miR-7	X		[1]
miR-9	X		[3–6]
miR-9*	X		[6]
miR-9-3p	X		[5]
miR-10a	X		[1,7,8]
miR-10b*		X	[1,6]
miR-10b		X	[9]
miR-10b	X		[10]
miR-15a		X	[11,12]
miR-15b	X		[13]
miR-17	X		[1]
miR-17-5p	X		[7]
miR-18a	X		[1,6]
miR-18a-3p	X		[5]
miR-18b	X		[1]
		X	[2]
miR-19b	X		[4]
miR-21		X	[9]
miR-23a	X		[7]
miR-23a*	X		[1]
		X	[6]
miR-23b		X	[1]
miR-23b-5p		X	[14]
miR-23c		X	[14]
miR-24-1*		X	[1]
miR-25	X		[7]
miR-25p	X		[15]
miR-26a	X		[4]
miR-26a1	X		[4]
miR-27a	X		[16,17]
miR-27b		X	[1]
miR-28	X		[7]
miR-29b		X	[1]
miR-29c		X	[18]
miR-30a-5p		X	[9]
miR-30a-3p		X	[9]
miR-30c		X	[19,20]
miR-30c2*	X		[1]
miR-31	X		[8]
		X	[18]
miR-32		X	[4]
miR-33a		X	[1]
miR-33b		X	[4]
miR-33b*	X		[1]
miR-34a	X		[7,21,22]

		X	[7,21–23]
miR-34b		X	[1]
miR-34b*		X	[1]
miR-34c		X	[24,25]
miR-34c-3p	X		[26]
miR-34c-5p		X	[1,14,26]
miR-95	X		[1,7]
miR-96	X		[1,3,6,8]
miR-98	X		[27]
miR-99a		X	[28,29]
miR-99a-3p		X	[15]
miR-99b		X	[8]
miR-100		X	[6,28]
miR-101		X	[1,30,31]
miR-103	X		[7,19]
miR-106a	X		[1,7,19]
miR-106b		X	[32,33]
miR-107	X		[7,19]
miR-123	X		[4]
miR-124a-1		X	[2]
miR-124a-2		X	[2]
miR-124a-3		X	[2]
miR-125a-3p	X		[1]
miR-125b-1	X		[4]
miR-125b-2		X	[13]
	X		[4]
miR-126		X	[34]
miR-127-3p		X	[1,6]
miR-129-2		X	[35]
miR-129-3p		X	[16]
miR-129-5p		X	[26]
miR-130b	X		[7]
		X	[36]
miR-132*		X	[1]
miR-133a		X	[1,13]
	X		[4]
miR-133b		X	[1,8,13]
miR-134	X		[1]
miR-135a*	X		[1]
miR-135a	X		[5,13]
miR-135b	X		[1,3,5,9,13]
miR-136		X	[1]
miR-136*		X	[1]
miR-137		X	[13,37]
miR-139-3p	X		[21]
miR-139-5p		X	[1,38]
miR-140-3p		X	[1]
miR-140-5p		X	[1]
miR-141	X		[3,6,7,13,39,40]
miR-142-3p		X	[1,11]
	X		[13]
miR-142-5p	X		[8,13]
		X	[1,11]
miR-143		X	[1,41]
	X		[4]
miR-143 *		X	[1]
miR-145		X	[1,36]
miR-145 *		X	[1]
miR-145a	X		[4]
miR-146	X		[4]
miR-146a	X		[6]
		X	[11]
miR-146b-5p	X		[21]
miR-148a		X	[2,20]
miR-148a-3p	X		[26]
miR-150*	X		[1]
miR-151	X		[7]

miR-152		X	[1,2,6,19,42,43]
miR-153	X		[17]
miR-155	X		[7,8,33]
miR-181a	X		[19,27,44]
miR-181c	X		[4]
miR-181c-3p	X		[15]
miR-182	X		[1,3,5,7,8,17,22,39,40,45]
miR-183	X		[1,3–5,7,22,45]
miR-183-5p	X		[46]
miR-184	X		[26]
		X	[47]
miR-185	X		[19]
		X	[18]
miR-186	X		[48,49]
miR-188-5p	X		[1]
miR-191	X		[19]
miR-193		X	[19]
miR-193a		X	[8]
miR-193a-3p	X		[21]
miR-193a-5p	X		[1]
miR-193b		X	[8]
miR-194	X		[7]
		X	[50–52]
miR-195		X	[1,9]
miR-196a-5p		X	[15,53]
miR-196b		X	[1,47]
miR-198	X		[1]
miR-199a-5p		X	[1]
miR-199b-5p		X	[1,5,6]
miR-199b-3p		X	[1,6]
miR-199b		X	[28]
miR-200		X	[54]
miR-200a	X		[1,6–8,13,22,28,45,55–57]
miR-200a-5p	X		[5]
miR-200a*	X		[1,28,39,55]
miR-200b	X		[1,3,8,13,55,56,58]
miR-200b*	X		[1,45,48]
miR-200c	X		[1,3,4,6–9,13,59,60]
		X	[61]
miR-202	X		[1]
		X	[62]
miR-203	X		[1,3,6–8,13]
miR-203		X	[63]
miR-204		X	[8,49]
	X		[14]
miR-204-5p		X	[64]
miR-205	X		[1,4,6,7,9,13,39,40,45,59,65]
miR-210	X		[3,6–8,13,19]
miR-214		X	[1,45]
miR-214*		X	[1]
miR-214-3p		X	[66]
miR-215	X		[7]
mIR-216b		X	[10]
miR-218	X		[5]
miR-221	X		[19,21]
miR-222	X		[48,49]
miR-222-3p	X		[67]
miR-223	X		[1,4,7,48,49]
miR-224	X		[1]
miR-299-3p		X	[1]
miR-299-5p		X	[1]
miR-301	X		[1]
miR-302a-5p		X	[68]
miR-325	X		[7]
miR-326	X		[7]
		X	[66]
miR-328-3p		X	[15]

miR-330	X		[7]
miR-330-3p	X		[1]
miR-337	X		[69]
miR-337-3p		X	[15]
miR-337-5p		X	[1,5]
miR 338-3p		X	[53]
miR-363	X		[8]]
miR 367-3p		X	[68]
miR-368		X	[8]
miR-369		X	[4]
miR-370		X	[5,6]
miR-371-5p	X		[1]
miR-375	X		[26]
miR-376a		X	[1,6]
miR-376c		X	[1,6,44]
miR-377		X	[1,44,58]
miR-379		X	[1]
miR-381		X	[1,6,70]
miR-382		X	[58]
miR-409		X	[4]
miR-410		X	[1,3,6,29,58]
miR-411		X	[1,22]
miR-421	X		[6]
miR-423	X		[4,19]
miR-424		X	[1,4,6,71]
miR-424*		X	[6]
miR-424-3p		X	[5]
miR-425	X		[1,4]
miR-429	X		[1,3,5,8,13]
miR-431		X	[4,6]
miR-432	X		[8]
		X	[6]
miR-449	X		[8]
miR-449a		X	[53]
miR-450a		X	[1]
miR-451		X	[4]
miR-455-3p		X	[1]
miR-455-5p		X	[1]
miR-483-5p	X		[1]
miR-487b		X	[22]
miR-494	X		[1]
miR-496		X	[4]
miR-497		X	[1,47]
miR-499	X		[9]
miR-501-5p	X		[1]
miR-503		X	[1,4,6,72]
miR-504		X	[13]
miR-505*	X		[1]
miR-513a-5p	X		[1]
miR-513b	X		[1]
miR-516		X	[4]
miR-516a-5p	X		[6]
miR-518c*	X		[1]
miR-518d-5p	X		[21]
miR-519a	X		[13]
miR-520-5p	X		[21]
miR-522	X		[21]
miR-526a	X		[21]
miR-542-3p		X	[1,6]
miR-542-5p		X	[1,5,49]
miR-557	X		[1]
miR-564	X		[1]
miR-572	X		[22]
miR-575	X		[73]
miR-596		X	[6]
miR-601	X		[4]
miR-605	X		[6]

miR-610		X	[6]
miR-614	X		[6]
miR-622	X		[1,22]
miR-623	X		[1]
miR-629*	X		[1]
miR-630	X		[1]
		X	[6]
miR-632		X	[6]
miR-650	X		[22]
miR-652	X		[1]
miR-654-3p		X	[1]
miR-663	X		[1]
miR-758		X	[5]
miR-760	X		[1]
		X	[6]
miR-765	X		[35]
miR-768-5p	X		[1]
miR-801	X		[1]
miR-873		X	[1]
miR-877	X		[1]
miR-892b	X		[1]
miR-888	X		[13]
miR-923	X		[1]
miR-936	X		[6]
miR-939	X		[1]
miR-944	X		[73]
mIR-1202	X		[53]
miR-1224-5p	X		[1]
miR-1225-5p	X		[1]
miR-1226	X		[1]
miR-1228	X		[3]
miR-1247		X	[5]
miR-1271		X	[74]
miR-1976		X	[11]
miR-3170		X	[11]
miR-4467		X	[26]
miR-5001-5p		X	[26]
miR-5787	X		[53]
miR-6749-5p	X		[53]
miR-6950-5p		X	[26]

Table S2. Expression profile of miRs within neoplastic endometrial tissue according to the associated ganglionic status.

Reference	Sample Type	Case Sample	Case	Control	miR Increased (Case vs. Control)	miR Decreased (Case vs. Control)
Canlorbe et al., 2016 [26]	Paraffin	Endometrioid endometrial cancers grade 1–2 (*n* = 36):	9 N+	27 N−		miR-23b-5p, -23c, -34c-5p, -34c-3p, -34b-5p, -184, -375
Li et al., 2016 [29]	Paraffin	Endometrioid endometrial cancers (*n* = 47):<br>-36 Grade 1–, 11 Grade 3<br>-34 FIGO I–II, 13 FIGO III–V	17 N+	30 N−	-	-
He et al., 2015 [44]	Paraffin	Endometrioid endometrial cancers (*n* = 47):<br>-32 Grade 1, 15 Grade 2–3<br>-38 FIGO I–II et 9 FIGO III–IV	5N+	42N−	miR-181a	-
Liu et al., 2014 [67]	−80 °C	Endometrial cancers (*n* = 75):<br>-36 Grade 1, 26 Grade 2, 13 Grade 3<br>-47 FIGO I, 16 FIGO II, 12 FIGO III	11 N+	64 N−	miR-222-3p	-
Tsukamoto et al., 2014 [9]	-	Endometrioid endometrial cancers (*n* = 28)	4 N+	21 N−	-	-
Bao et al., 2013 [64]	−80 °C	Endometrial cancers (*n* = 71):<br>-57 endometrioid, 14 others<br>-32 Grade 1, 18 Grade 2, 7 Grade 3<br>-60 FIGO I, 7 FIGO II, 4 FIGO III	8 N+	63 N−	-	miR-204-5p
Zhang et al., 2013 [41]	Paraffin	Endometrial cancers (*n* = 107):<br>-85 endometrioid, 22 others<br>-30 Grade 1, 39 Grade 2, 16 Grade 3<br>-18 LVSI+, 87 LVSI–<br>-74 FIGO I, 17 FIGO II, 13 FIGO III, 1 FIGO IV	6 N+	42 N−	-	-
Torres et al., 2013 [3]	Paraffin<br>−80 °C	Endometrioid endometrial cancers (*n* = 77):<br>-29 Grade 1, 30 Grade 2, 18 Grade 3	15 N+	29 N−	miR-200a* /miR-203/miR-429	-
Torres et al., 2012 [28]	Paraffin<br>−80 °C	Endometrioid endometrial cancers (*n* = 77):<br>-29 Grade 1, 30 Grade 2, 18 Grade 3<br>-50 FIGO I, 5 FIGO II, 15 FIGO III, 2 FIGO IV	29 N+	15 N−	-	-
Cohn et al., 2010 [4]	Paraffin	Endometrial cancers (*n* = 141):<br>-121 endometriod FIGO I,<br>-3 endometrioid FIGO III,<br>-7 serous FIGO III,<br>-4 endometrioid FIGO IV,	20 FIGO III-IV	121 FIGO I	miR-145a, -10b, -123, 26a, 125b1, -125b2, -143, -133a, -26a1	-

		-6 serous FIGO IV				
Hiroki et al., 2010 [1]	−80 °C	Serous adenocarcinoma (n = 21):<br>-8 FIGO I, 2 FIGOII, 3 FIGOIII, 8 FIGO IV<br>-5 LVSI+, 16 LVSI−	2 N+	13 N−	-	-
Chung et al., 2009 [7]	−80 °C	Endometrioid endometrial cancers (n = 30):<br>-25 FIGOI–II, 5 FIGO III<br>-19 grade 1, 11 grade 2	3 N+	27 N−	miR-10a, -34a, -95	-

FIGO: International Federation of Gynecology et Obstetrics, LVSI: Lympho-vascular emboli, N: ganglionic status

Table S3. Relationship between plasma / serum miRs and endometrial cancer.

Reference	Sample	Case	Control	miR Increased (Case vs. Control)	miR Decreased (Case vs. Control)
Montagnana et al., 2017 [49]	Plasma	Endometrial cancers (n = 48)	Healthy patients (n = 28)	miR-186, miR-222, miR-223	miR-204
Jia et al., 2013 [48]	Serum	Endometrioid endometrial cancers (n = 33)	Healthy patients (n = 42)	miR-222, miR-223, miR-186 and miR-204	-
Torres et al., 2013 [3]	Plasma	Endometrioid endometrial cancers (n = 34)	Healthy patients (n = 14)	miR-92a, miR-141, miR-200a, miR-203, miR-449a, miR-1228 and miR-1290	miR-9 et miR-301b
Torres et all., 2012 [28]	Plasma	Endometrioid endometrial cancers (n = 48)	Healthy patients (n = 14)	miR-99a, miR-100 et miR-199b	-
Tsukamoto et al., 2014 [9]	Plasma	Endometrioid endometrial cancers (n = 12)	Healthy patients (n = 12)	miR-135b, miR-205	miR-30a-3p, miR-21
Wang et al., 2014 [75]	Plasma	Endometrioid endometrial cancers (n = 40)	Healthy patients (n = 53)	miR-27a, miR-15b, miR-143, miR-223	miR-1179, miR-4638-3p, miR-4665-5p, miR-3145-5p, miR-4502

References

1. Hiroki, E.; Akahira, J.-I.; Suzuki, F.; Nagase, S.; Ito, K.; Suzuki, T.; Sasano, H.; Yaegashi, N. Changes in microRNA expression levels correlate with clinicopathological features and prognoses in endometrial serous adenocarcinomas. *Cancer Sci.* **2010**, *101*, 241–249.
2. Pavicic, W.; Perkiö, E.; Kaur, S.; Peltomäki, P. Altered methylation at MicroRNA-associated CpG islands in hereditary and sporadic carcinomas: A methylation-specific multiplex ligation-dependent probe amplification (MS-MLPA)-based approach. *Mol. Med.* **2011**, *17*, 726–735.
3. Torres, A.; Torres, K.; Pesci, A.; Ceccaroni, M.; Paszkowski, T.; Cassandrini, P.; Zamboni, G.; Maciejewski, R. Diagnostic and prognostic significance of miRNA signatures in tissues and plasma of endometrioid endometrial carcinoma patients. *Int. J. Cancer* **2013**;*132*, 1633–1645, doi:10.1002/ijc.27840.
4. Cohn, D.E.; Fabbri, M.; Valeri, N.; Alder, H.; Ivanov, I.; Liu, C.-G.; Croce, C.M.; Resnick, K.E. Comprehensive miRNA profiling of surgically staged endometrial cancer. *Am. J. Obstet. Gynecol.* **2010**, *202*, 656.e1–656.e8.
5. Jurcevic, S.; Klinga-Levan, K.; Olsson, B.; Ejeskär, K. Verification of microRNA expression in human endometrial adenocarcinoma. *BMC Cancer* **2016**, *16*, 227.
6. Snowdon, J.; Zhang, X.; Childs, T.; Tron, V.A.; Feilotter, H. The MicroRNA-200 family is upregulated in endometrial carcinoma. *PLOS ONE* **2011**, *6*, e22828.
7. Chung, T.K.; Cheung, T.-H.; Huen, N.-Y.; Wong, K.W.; Lo, K.W.; Yim, S.-F.; Siu, N.S.; Wong, Y.-M.; Tsang, P.-T.; Pang, M.-W.; et al. Dysregulated microRNAs and their predicted targets associated with endometrioid endometrial adenocarcinoma in Hong Kong women. *Int. J. Cancer* **2009**, *124*, 1358–1365.
8. Wu, W.; Lin, Z.; Zhuang, Z.; Liang, X. Expression profile of mammalian microRNAs in endometrioid adenocarcinoma. *Eur. J. Cancer Prev.* **2009**, *18*, 50–55.
9. Tsukamoto, O.; Miura, K.; Mishima, H.; Abe, S.; Kaneuchi, M.; Higashijima, A.; Miura, S.; Kinoshita, A.; Yoshiura, K.-I.; Masuzaki, H. Identification of endometrioid endometrial carcinoma-associated microRNAs in tissue and plasma. *Gynecol. Oncol.* **2014**, *132*, 715–721.
10. Xie, P.; Cao, H.; Li, Y.; Wang, J.; Cui, Z. Knockdown of lncRNA CCAT2 inhibits endometrial cancer cells growth and metastasis via sponging miR-216b. *Cancer Biomark.* **2017**, *21*, 123–133.
11. Wang, Y.; Xu, M.; Yang, Q. A six-microRNA signature predicts survival of patients with uterine corpus endometrial carcinoma. *Curr. Probl. Cancer* **2019**, *43*, 167–176, doi:10.1016/j.currproblcancer.2018.02.002.
12. Wang, Z.M.; Wan, X.H.; Sang, G.Y.; Zhao, J.D.; Zhu, Q.Y.; Wang, D.M. miR-15a-5p suppresses endometrial cancer cell growth via Wnt/β-catenin signaling pathway by inhibiting WNT3A. *Eur. Rev. Med. Pharmacol. Sci.* **2017**, *21*, 4810–4818.
13. Devor, E.J.; Hovey, A.M.; Goodheart, M.J.; Ramachandran, S.; Leslie, K.K. microRNA expression profiling of endometrial endometrioid adenocarcinomas and serous adenocarcinomas reveals profiles containing shared, unique and differentiating groups of microRNAs. *Oncol. Rep.* **2011**, *26*, 995–1002.
14. Canlorbe, G.; Castela, M.; Bendifallah, S.; Wang, Z.; Lefevre, M.; Chabbert-Buffet, N.; Aractingi, S.; Darai, E.; Méhats, C.; Ballester, M. *Histol. Histopathol.* **2017**, *32*, 941–950, doi:10.14670/HH-11-859.
15. Xiong, H.; Li, Q.; Liu, S.; Wang, F.; Xiong, Z.; Chen, J.; Chen, H.; Yang, Y.; Tan, X.; Luo, Q.; et al. Integrated microRNA and mRNA transcriptome sequencing reveals the potential roles of miRNAs in stage I endometrioid endometrial carcinoma. *PLOS ONE* **2014**, *9*, e110163.
16. Zhang, R.; He, Y.; Zhang, X.; Xing, B.; Sheng, Y.; Lu, H.; Wei, Z. Estrogen receptor-regulated microRNAs contribute to the BCL2/BAX imbalance in endometrial adenocarcinoma and precancerous lesions. *Cancer Lett.* **2012**, *314*, 155–165.
17. Lin, X.; Qiu, J.; Hua, K. Long non-coding RNAs as emerging regulators of epithelial to mesenchymal transition in gynecologic cancers. *Biosci. Trends* **2018**, *12*, 342–353.
18. Ushakov, D.S.; Dorozhkova, A.S.; Babayants, E.V.; Ovchinnikov, V.Y.; Kushlinskii, D.N.; Adamyan, L.V.; Gulyaeva, L.F.; Kushlinskii, N.E. Expression of microRNA Potentially Regulated by AhR and CAR in Malignant Tumors of the Endometrium. *Bull. Exp. Boil. Med.* **2018**, *165*, 688–691.
19. Boren, T.; Xiong, Y.; Hakam, A.; Wenham, R.; Apte, S.; Wei, Z.; Kamath, S.; Chen, D.-T.; Dressman, H.; Lancaster, J.M. MicroRNAs and their target messenger RNAs associated with endometrial carcinogenesis. *Gynecol. Oncol.* **2008**, *110*, 206–215.

20. Aprelikova, O.; Palla, J.; Hibler, B.; Yu, X.; Greer, Y.E.; Yi, M.; Stephens, R.; Maxwell, G.L.; Jazaeri, A.; Risinger, J.I.; et al. Silencing of miR-148a in cancer-associated fibroblasts results in WNT10B-mediated stimulation of tumor cell motility. *Oncogene* **2013**, *32*, 3246–3253, doi:10.1038/onc.2012.351.
21. Wang, Z.; Wang, W.; Huang, K.; Wang, Y.; Li, J.; Yang, X. MicroRNA-34a inhibits cells proliferation and invasion by downregulating Notch1 in endometrial cancer. *Oncotarget* **2017**, *8*, 111258–111270.
22. Ratner, E.S.; Tuck, D.; Richter, C.; Nallur, S.; Patel, R.M.; Schultz, V.; Hui, P.; Schwartz, P.E.; Rutherford, T.J.; Weidhaas, J.B. MicroRNA signatures differentiate uterine cancer tumor subtypes. *Gynecol. Oncol.* **2010**, *118*, 251–257.
23. Schirmer, U.; Doberstein, K.; Rupp, A.-K.; Bretz, N.P.; Wuttig, D.; Kiefel, H.; Breunig, C.; Fiegl, H.; Müller-Holzner, E.; Zeillinger, R.; et al. Role of miR-34a as a suppressor of L1CAM in endometrial carcinoma. *Oncotarget* **2014**, *5*, 462–472.
24. Li, F.; Chen, H.; Huang, Y.; Zhang, Q.; Xue, J.; Liu, Z.; Zheng, F. miR-34c plays a role of tumor suppressor in HEC-1-B cells by targeting E2F3 protein. *Oncol. Rep*. **2015**, *33*, 3069–3074. doi:10.3892/or.2015.3894.
25. Jiang, L.; Meng, W.; Zeng, J.; Hu, H.; Lu, L. MiR-34c oligonucleotide enhances chemosensitivity of Ishikawa cell to cisplatin by inducing apoptosis. *Cell Boil. Int.* **2013**, *37*, 577–583.
26. Canlorbe, G.; Wang, Z.; Laas, E.; Bendifallah, S.; Castela, M.; Lefèvre, M.; Chabbert-Buffet, N.; Daraï, E.; Aractingi, S.; Méhats, C.; et al. Identification of microRNA expression profile related to lymph node status in women with early-stage grade 1–2 endometrial cancer. *Mod. Pathol.* **2016**, *29*, 391–401.
27. Panda, H.; Chuang, T.-D.; Luo, X.; Chegini, N. Endometrial miR-181a and miR-98 expression is altered during transition from normal into cancerous state and target PGR, PGRMC1, CYP19A1, DDX3X, and TIMP3. *J. Clin. Endocrinol. Metab.* **2012**, *97*, E1316–E1326.
28. Torres, A.; Torres, K.; Pesci, A.; Ceccaroni, M.; Paszkowski, T.; Cassandrini, P.; Zamboni, G.; Maciejewski, R. Deregulation of miR-100, miR-99a and miR-199b in tissues and plasma coexists with increased expression of mTOR kinase in endometrioid endometrial carcinoma. *BMC Cancer* **2012**, *12*, 369.
29. Li, Y.; Zhang, Z.; Zhang, X.; Lin, Y.; Luo, T.; Xiao, Z.; Zhou, Q. A dual PI3K/AKT/mTOR signaling inhibitor miR-99a suppresses endometrial carcinoma. *Am. J. Transl. Res.* 2016, *8*, 719–731.
30. Liu, Y.; Li, H.; Zhao, C.; Jia, H. MicroRNA-101 inhibits angiogenesis via COX-2 in endometrial carcinoma. *Mol. Cell. Biochem.* **2018**, *448*, 61–69.
31. Zhang, S.; Wang, M.; Li, Q.; Zhu, P. MiR-101 reduces cell proliferation and invasion and enhances apoptosis in endometrial cancer via regulating PI3K/Akt/mTOR. *Cancer Biomark.* **2017**, *21*, 179–186.
32. Huang, C.; Hu, G. Shikonin suppresses proliferation and induces apoptosis in endometrioid endometrial cancer cells via modulating miR-106b/PTEN/AKT/mTOR signaling pathway. *Biosci. Rep.* **2018**, *38*, 3055–3060.
33. Choi, C.H.; Park, Y.-A.; Choi, J.-J.; Song, T.; Song, S.Y.; Lee, Y.-Y.; Lee, J.-W.; Kim, T.-J.; Kim, B.-G.; Bae, D.-S. Angiotensin II type I receptor and miR-155 in endometrial cancers: Synergistic antiproliferative effects of anti-miR-155 and losartan on endometrial cancer cells. *Gynecol. Oncol.* **2012**, *126*, 124–131.
34. Zhao, X.; Zhu, D.; Lu, C.; Yan, D.; Li, L.; Chen, Z. MicroRNA-126 inhibits the migration and invasion of endometrial cancer cells by targeting insulin receptor substrate 1. *Oncol. Lett*. **2016**, *11*, 1207–1212, doi:10.3892/ol.2015.4001.
35. Huang, Y.-W.; Liu, J.C.; Deatherage, D.E.; Luo, J.; Mutch, D.G.; Goodfellow, P.J.; Miller, D.S.; Huang, T.H.-M. Epigenetic repression of microRNA-129-2 leads to overexpression of SOX4 oncogene in endometrial cancer. *Cancer Res.* **2009**, *69*, 9038–9046.
36. Dong, P.; Karaayvaz, M.; Jia, N.; Kaneuchi, M.; Hamada, J.; Watari, H.; Sudo, S.; Ju, J.; Sakuragi, N. Mutant p53 gain-of-function induces epithelial-mesenchymal transition through modulation of the miR-130b-ZEB1 axis. *Oncogene* **2013**, *32*, 3286–3295, doi:10.1038/onc.2012.334.
37. Zhang, W.; Chen, J.H.; Shan, T.; Aguilera-Barrantes, I.; Wang, L.S.; Huang, T.H.; Rader, J.S.; Sheng, X.; Huang, Y.W. miR-137 is a tumor suppressor in endometrial cancer and is repressed by DNA hypermethylation. *Lab. Invest*. **2018**, *98*, 1397–1407, doi:10.1038/s41374-018-0092-x.
38. Liu, J.; Li, C.; Jiang, Y.; Wan, Y.; Zhou, S.; Cheng, W. Tumor-suppressor role of miR-139-5p in endometrial cancer. *Cancer Cell Int.* **2018**, *18*, 51.
39. Lee, T.S.; Jeon, H.W.; Kim, Y.B.; Kim, Y.A.; Kim, M.A.; Kang, S.B. Aberrant MicroRNA expression in endometrial carcinoma using formalin-fixed paraffin-embedded (FFPE) tissues. *PLOS ONE* **2013**, *8*, e81421.

40. Kottaridi, C.; Spathis, A.; Margari, N.; Koureas, N.; Terzakis, E.; Chrelias, C.; Pappas, A.; Bilirakis, E.; Pouliakis, A.; Panayiotides, I.J.; et al. Evaluation analysis of miRNAs overexpression in liquid-based cytology endometrial samples. *J. Cancer* **2017**, *8*, 2699–2703.
41. Zhang, X.; Dong, Y.; Ti, H.; Zhao, J.; Wang, Y.; Li, T.; Zhang, B. Down-regulation of miR-145 and miR-143 might be associated with DNA methyltransferase 3B overexpression and worse prognosis in endometrioid carcinomas. *Hum. Pathol.* **2013**, *44*, 2571–2580.
42. Tsuruta, T.; Kozaki, K.-I.; Uesugi, A.; Furuta, M.; Hirasawa, A.; Imoto, I.; Susumu, N.; Aoki, D.; Inazawa, J. miR-152 is a tumor suppressor microRNA that is silenced by DNA hypermethylation in endometrial cancer. *Cancer Res.* **2011**, *71*, 6450–6462.
43. Xie, D.; Liang, Y.; Su, Y.; An, Y.; Qu, P. miR-152 inhibits proliferation of human endometrial cancer cells via inducing G2/M phase arrest by suppressing CDC25B expression. *Biomed. Pharmacother.* **2018**, *99*, 299–305.
44. Zeng, S.; Zhou, Z.-W.; He, Z.-X.; Zhou, S.-F.; He, S.-M.; He, Z. Hsa-microRNA-181a is a regulator of a number of cancer genes and a biomarker for endometrial carcinoma in patients: a bioinformatic and clinical study and the therapeutic implication. *Drug Des. Dev. Ther.* **2015**, *9*, 1103–75.
45. Lee, H.; Choi, H.J.; Kang, C.S.; Lee, H.J.; Lee, W.S.; Park, C.S. Expression of miRNAs and PTEN in endometrial specimens ranging from histologically normal to hyperplasia and endometrial adenocarcinoma. *Mod. Pathol.* **2012**, *25*, 1508–1515.
46. Yan, H.; Sun, B.; Zhang, Y.; Li, Y.; Huang, C.; Feng, F.; Li, C. Upregulation of miR-183-5p is responsible for the promotion of apoptosis and inhibition of the epithelial-mesenchymal transition, proliferation, invasion and migration of human endometrial cancer cells by downregulating Ezrin. *Int. J. Mol. Med.* **2018**, *42*, 2469–2480.
47. De Foucher, T.; Sbeih, M.; Uzan, J.; Bendifallah, S.; Lefevre, M.; Chabbert-Buffet, N.; Aractingi, S.; Uzan, C.; Alsalam, I.A.; Mitri, R.; et al. Identification of micro-RNA expression profile related to recurrence in women with ESMO low-risk endometrial cancer. *J. Transl. Med*. **2018**, *16*, 131, doi:10.1186/s12967-018-1515-6.
48. Jia, W.; Wu, Y.; Zhang, Q.; Gao, G.; Zhang, C.; Xiang, Y. Identification of four serum microRNAs from a genome-wide serum microRNA expression profile as potential non-invasive biomarkers for endometrioid endometrial cancer. *Oncol. Lett.* **2013**, *6*, 261–267.
49. Montagnana, M.; Benati, M.; Danese, E.; Giudici, S.; Perfranceschi, M.; Ruzzenenete, O.; Salvagno, G.L.; Bassi, A.; Gelati, M.; Paviati, E.; et al. Aberrant MicroRNA expression in patients with endometrial cancer. *Int. J. Gynecol. Cancer* **2017**, *27*, 459–466.
50. Zhai, H.; Karaayvaz, M.; Dong, P.; Sakuragi, N.; Ju, J. Prognostic significance of miR-194 in endometrial cancer. *Biomark. Res.* **2013**, *1*, 12.
51. Dong, P.; Kaneuchi, M.; Watari, H.; Hamada, J.; Sudo, S.; Ju, J.; Sakuragi, N. MicroRNA-194 inhibits epithelial to mesenchymal transition of endometrial cancer cells by targeting oncogene BMI-1. *Mol. Cancer* **2011**, *10*, 99.
52. Gong, B.; Yue, Y.; Wang, R.; Zhang, Y.; Jin, Q.; Zhou, X. Overexpression of microRNA-194 suppresses the epithelial–mesenchymal transition in targeting stem cell transcription factor Sox3 in endometrial carcinoma stem cells. *Tumor Boil.* **2017**, *39*.
53. Fan, Y.; Xu, W.; Meng, Y.; Fang, D.; Wang, J.; Chen, H. Exploration of miR-1202 and miR-196a in human endometrial cancer based on high throughout gene screening analysis. *Oncol. Rep.* **2017**, *37*, 3493–3501.
54. Bai, J.-X.; Yan, B.; Zhao, Z.-N.; Xiao, X.; Qin, W.-W.; Zhang, R.; Jia, L.-T.; Meng, Y.-L.; Jin, B.-Q.; Fan, D.-M.; et al. Tamoxifen represses miR-200 MicroRNAs and promotes epithelial-to-mesenchymal transition by up-regulating c-Myc in endometrial carcinoma cell lines. *Endocrinology* **2013**, *154*, 635–645.
55. Yoneyama, K.; Ishibashi, O.; Kawase, R.; Kurose, K.; Takeshita, T. miR-200a, miR-200b and miR-429 are onco-miRs that target the PTEN gene in endometrioid endometrial carcinoma. *Anticancer. Res.* **2015**, *35*, 1401–1410.
56. Lu, R.-L.; Li, J.-X.; Rong, L.-J.; Wu, Q. MiR-200a and miR-200b target PTEN to regulate the endometrial cancer cell growth in vitro. *Asian Pac. J. Trop. Med.* **2017**, *10*, 498–502.
57. Shi, W.; Wang, X.; Ruan, L.; Fu, J.; Liu, F.; Qu, J. MiR-200a promotes epithelial-mesenchymal transition of endometrial cancer cells by negatively regulating FOXA2 expression. *Die Pharm.* **2017**, *72*, 694–699.
58. Mehlich, D.; Garbicz, F.; Domosud, Z.; Paskal, W.; Rak, B.; Marczewska, J.M.; Włodarski, P.K. Post-transcriptional regulation of MMP16 and TIMP2 expression via miR-382, miR-410 and miR-200b in endometrial cancer. *Cancer Genomics Proteomics* **2017**, *14*, 389–401.

59. Karaayvaz, M.; Zhang, C.; Liang, S.; Shroyer, K.R.; Ju, J. Prognostic significance of miR-205 in endometrial cancer. *PLOS ONE* **2012**, *7*, e35158.
60. Wilczynski, M.; Danielska, J.; Domanska-Senderowska, D.; Dzieniecka, M.; Szymańska, B.; Malinowski, A. Association of microRNA-200c expression levels with clinicopathological factors and prognosis in endometrioid endometrial cancer. *Acta Obstet. Gynecol. Scand.* **2018**, *97*, 560–569.
61. Li, F.; Liang, A.; Lv, Y.; Liu, G.; Jiang, A.; Liu, P. MicroRNA-200c inhibits epithelial-mesenchymal transition by targeting the BMI-1 gene through the phospho-AKT pathway in endometrial carcinoma cells in vitro. *Med. Sci. Monit.* **2017**, *23*, 5139–5149.
62. Deng, X.; Hou, C.; Liang, Z.; Wang, H.; Zhu, L.; Xu, H. miR-202 suppresses cell proliferation by targeting FOXR2 in endometrial adenocarcinoma. *Dis. Mark.* **2017**, *2017*, 1–8.
63. Huang, Y.-W.; Kuo, C.-T.; Chen, J.-H.; Goodfellow, P.J.; Huang, T.H.-M.; Rader, J.S.; Uyar, D.S. Hypermethylation of miR-203 in endometrial carcinomas. *Gynecol. Oncol.* **2014**, *133*, 340–345.
64. Bao, W.; Wang, H.-H.; Tian, F.-J.; He, X.-Y.; Qiu, M.-T.; Wang, J.-Y.; Zhang, H.-J.; Wang, L.-H.; Wan, X.-P. A TrkB–STAT3–miR-204-5p regulatory circuitry controls proliferation and invasion of endometrial carcinoma cells. *Mol. Cancer* **2013**, *12*, 155.
65. Zhuo, Z.; Yu, H. miR-205 inhibits cell growth by targeting AKT-mTOR signaling in progesterone-resistant endometrial cancer Ishikawa cells. *Oncotarget* **2017**, *8*, 28042–28051.
66. Liu, W.; Zhang, B.; Xu, N.; Wang, M.J.; Liu, Q. miR-326 regulates EMT and metastasis of endometrial cancer through targeting TWIST1. *Eur. Rev. Med. Pharmacol. Sci.* **2017**;*21*, 3787–3793.
67. Liu, B.; Che, Q.; Qiu, H.; Bao, W.; Chen, X.; Lü, W.; Li, B.; Wan, X. Elevated MiR-222-3p Promotes Proliferation and Invasion of Endometrial Carcinoma via Targeting ERα. *PLOS ONE* **2014**, *9*, e87563.
68. Ma, J.; Li, D.; Kong, F.-F.; Yang, D.; Yang, H.; Ma, X.-X. miR-302a-5p/367-3p-HMGA2 axis regulates malignant processes during endometrial cancer development. *J. Exp. Clin. Cancer Res.* **2018**, *37*, 19.
69. Cai, Y.; He, T.; Liang, L.; Zhang, X.; Yuan, H. Upregulation of microRNA-337 promotes the proliferation of endometrial carcinoma cells via targeting PTEN. *Mol. Med. Rep*. **2016**;*13*, 4827–4834, doi:10.3892/mmr.2016.5134.
70. Tu, C.; Wang, F.; Wan, J. MicroRNA-381 inhibits cell proliferation and invasion in endometrial carcinoma by targeting the IGF-1R. *Mol. Med. Rep*. **2018**, *17*, 4090–4098, doi:10.3892/mmr.2017.8288.
71. Li, Q.; Qiu, X.-M.; Li, Q.-H.; Wang, X.-Y.; Li, L.; Xu, M.; Dong, M.; Xiao, Y.-B. MicroRNA-424 may function as a tumor suppressor in endometrial carcinoma cells by targeting E2F7. *Oncol. Rep.* **2015**, *33*, 2354–2360.
72. Xu, Y.-Y.; Wu, H.-J.; Ma, H.-D.; Xu, L.-P.; Huo, Y.; Yin, L.-R. MicroRNA-503 suppresses proliferation and cell-cycle progression of endometrioid endometrial cancer by negatively regulating cyclin D1. *FEBS J.* **2013**, *280*, 3768–3779.
73. He, Z.; Xu, H.; Meng, Y.; Kuang, Y. miR-944 acts as a prognostic marker and promotes the tumor progression in endometrial cancer. *Biomed. Pharmacother.* **2017**, *88*, 902–910.
74. Li, L.; Qu, Y.W.; Li, Y.P. Over-expression of miR-1271 inhibits endometrial cancer cells proliferation and induces cell apoptosis by targeting CDK1. *Eur. Rev. Med. Pharmacol. Sci*. **2017**, *21*, 2816–2822.
75. Wang, L.; Chen, Y.-J.; Xu, K.; Xu, H.; Shen, X.-Z.; Tu, R.-Q. Circulating microRNAs as a fingerprint for endometrial endometrioid adenocarcinoma. *PLOS ONE* **2014**, *9*, e110767.

© 2019 by the authors. Licensee MDPI, Basel, Switzerland. This article is an open access article distributed under the terms and conditions of the Creative Commons Attribution (CC BY) license (http://creativecommons.org/licenses/by/4.0/).
